# Myocardial late gadolinium enhancement in systemic lupus erythematosus as a marker of chronic inflammation

**DOI:** 10.1186/1532-429X-15-S1-P121

**Published:** 2013-01-30

**Authors:** Maria Fernanda Braggion Santos, Mohamed A Abdelrazek, Evangelos Giannitsis, Florian Andre

**Affiliations:** 1 Universitatsklinikum Heidelberg, Heidelberg, Germany; 2 Radiology, Cairo university, Faculty of Medicine, Cairo, Egypt; 3 School of Medicine of Ribeirao Preto University of Sao Paulo, Sao Paulo, Brazil

## Background

Cardiac involvement of patients with systemic lupus erythematosus (SLE) is one of the main complications contributing to morbidity and mortality, however a significant portion of patients presents with subclinical disease. Non-invasive contrast-enhanced cardiovascular magnetic resonance (CMR) imaging is a well-established diagnostic tool to identify myocardial tissue alterations and morphological and functional changes. In this study, we aimed to assess cardiac abnormalities in SLE patients using LGE-CMR and, furthermore, the relation of serological inflammatory biomarkers to the cardiac LGE-CMR findings.

## Methods

We studied 27 SLE patients (24 females, 45 ± 13 years) and 30 healthy age-matched volunteers who served as a control group (27 females, 44 ± 11 years). Cine MRI with 32 channel image acquisition and vector-ECG gated short axis, two and four chamber cine slices with parallel image acquisition covering the entire left ventricle (LV) were acquired using a regular SSFP sequence on a 1.5T Whole Body MRI scanner (Achieva 1.5T, Philips Medical Systems). LGE-CMR (GadoliniumDTPA: 0.2 mmol/kg, Magnevist) was performed and analyzed by two blinded experienced observers. Analysis of the presence and distribution of LGE forms was compared to serum levels of anti-ds DNA, C- reactive protein as markers of disease activity and inflammation. Groups were compared using the Student's t-test or the Mann-Whitney test and Fisher's exact test. P-values ≤ 0.05 were considered statistically significant.

## Results

**Figure 1:**
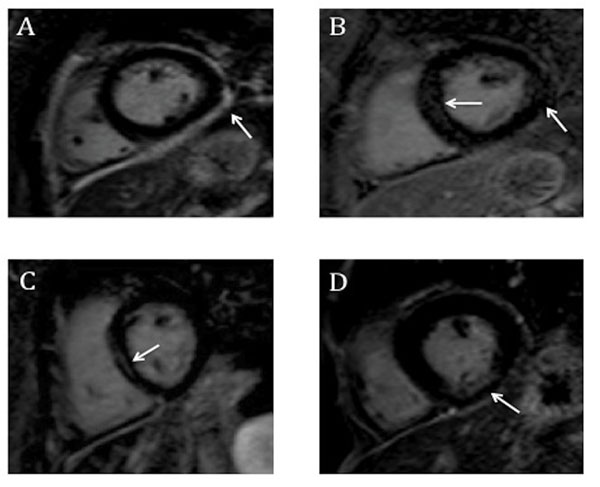
LGE short axis images in SLE patients with different patterns of cardiac involvement. Figures 1A to 1C illustrate infract-atypical patters: epi-myocardial/pericardial (A), patchy (B) and midwall (C). Figure 1D shows a 53 years-old man with a scar involving the subendocardium with no history of a previous event (silent myocardial infarction).

Nineteen SLE patients (70%) showed LV myocardial LGE (14 patients with infarct-atypical pattern, 2 patients with infarct-typical and 3 patients with mixed pattern, (Figure 1). Amongst the 5 patients with infarct-typical LGE pattern, 2 had antiphospholipid syndrome whereas 3 cases were associated with accelerated atherosclerosis. The presence of LGE was associated with CRP levels higher than 2 mg/l (p = 0,03), independent of the pattern of cardiac involvement (Table 1).

**Table 1:** Clinical characteristics and disease features in SLE patients according to the presence or absence of myocardial involvement revealed on LGE images.

	LGE positive (n = 19)	LGE negative (n = 8)	p value
Clinical and laboratorial features			

Age, years	43.8 ± 12.4	48.9 ± 13.2	n.s.
SLE duration, years	13.6 ± 5.1	15.2 ± 10.6	n.s.
SLEDAI score (>4), no. (%)	5 (26)	0 (0)	n.s.
Cardiovascular symptoms, no. (%)	4 (21)	4 (50)	n.s.
Antiphospholipid syndrome, no. (%)	5 (26)	1 (12.5)	n.s.
Anti-ds DNA antibodies (10 µl/l), no. (%)	14 (74)	6 (75)	n.s.
CRP* (>2 mg/l), no.(%)	12 (63)	1 (12.5)	0.03
MDRD** (ml/min/1.73 m^2^)	81.1 ± 39.3	65.3 ± 27.7	n.s.

CRP*: C-reative protein

** Estimated glomerular filtration rate

## Conclusions

Presence of myocardial LGE in SLE patients is a common finding and may be associated with a chronic and exacerbated inflammatory process. Taking into consideration the significant cardiovascular morbidity and mortality in SLE patients, and the difficulty in the early detection of cardiac manifestations, CMR could serve as a non-invasive diagnostic tool to assess cardiac SLE involvement and to aid in the cardiovascular risk stratification of these patients.

## Funding

none

